# Business intelligence for detecting possible surgical site infections from post-cesarean section operation with a focus on antibiotic prescriptions in Ramathibodi Hospital, Thailand

**DOI:** 10.1017/ash.2025.10224

**Published:** 2025-11-17

**Authors:** Thipakorn Pornmee, Kumthorn Malathum, Chonnamet Techasaensiri, Patratorn Kunakorntham, Thanomvong Muntajit

**Affiliations:** 1 Infection Prevention and Control Unit, Faculty of Medicine Ramathibodi Hospital, Mahidol University, Bangkok, Thailand; 2 Division of Infectious Diseases, Department of Medicine, Faculty of Medicine Ramathibodi Hospital, Mahidol University, Bangkok, Thailand; 3 Division of Infectious Diseases, Department of Pediatrics, Faculty of Medicine Ramathibodi Hospital, Mahidol University, Bangkok, Thailand; 4 Information Technology Department, Faculty of Medicine Ramathibodi Hospital, Mahidol University, Bangkok, Thailand

## Abstract

**Objective:**

To evaluate the effectiveness of postcaesarean infection surveillance using the Power Business Intelligence (BI) program, focusing on antibiotic prescriptions. Second, to compare the workload between the traditional and new approaches.

**Design:**

A diagnostic accuracy and workload evaluation.

**Setting:**

A tertiary care university hospital in metropolitan Bangkok, Thailand.

**Participants:**

All patients who underwent cesarean section between January 1, 2019, and September 30, 2020.

**Method:**

ICD-10 diagnoses, microbiological cultures, and postcesarean section antibiotic prescriptions in 3,243 medical records were captured by the Power BI program to detect surgical site infections (SSIs). All cases underwent conventional surveillance, which independently performed by infection control nurses. All patients were under surveillance until 45 days after surgery to capture delayed SSI diagnosis. SSIs were compared with sensitivity and specificity used to evaluate the new method. The Wilcoxon signed-rank test was employed to compare workload differences between the two methods in a paired-sample design.

**Results:**

The findings demonstrated the high sensitivity (100%) (95% CI: 66.4–100%) and specificity (93%) (95% CI: 90.5–95.4%) of the Power BI method when focusing on antibiotic prescriptions between 8- and 45-days postoperation. Additionally, the Power BI infection monitoring system significantly reduced the number of cases requiring review from 452 to 39 patients (a 91% reduction), indicating a substantial decrease in workload after implementation (*P* < .001).

**Conclusion:**

This antibiotic prescription-based, semi-automated surveillance program significantly reduced workload, demonstrating its potential to enhance infection monitoring in postcesarean section cases.

## Background

Infections occurring at surgical sites are of paramount concern. It can range from mild to severe, depending on various factors, such as the type of surgery, the extent of the infection, and the overall health of the individual. While some surgical site infections (SSIs) may only cause minor discomfort or delayed healing, others can lead to serious complications such as deep tissue infections, abscesses, or even systemic infections that spread throughout the body.

SSIs contribute significantly to maternal morbidity, especially when they occur in the pelvic cavity or develop as abscesses at the surgical site. Additionally, these infections have financial implications for patients, their families,^1,2^ and the healthcare system. Previous research has demonstrated that SSIs correlate with attributable mortality rates reaching 8–10%, prolonged hospitalization of approximately 15–18 days, and additional costs per case ranging from €9,560 to €33,500 in European healthcare settings.^1^ In the United States, healthcare-associated infections, including SSIs, result in an average additional cost of approximately $18,200 per case and extended hospital stays of around 9 days. ^2^

Postcesarean section undergoes meticulous scrutiny and infection monitoring by the infection prevention and control committee. The National Healthcare Safety Network (NHSN) recommends surveillance for SSIs following cesarean sections for a period of 30 days postoperatively.^3^ However, this surveillance process requires detailed protocols that are highly labor- and time-intensive.

To increase efficiency, healthcare facilities are increasingly adopting technology-driven solutions, such as electronic health records and automated surveillance systems, to streamline the process and improve detection of SSIs while reducing workload for staff.^4^

Business intelligence (BI) software constitutes a vital component of information technology employed for the aggregation, analysis, synthesis, and comprehension of data obtained from various business domains, facilitating enhanced decision-making processes within organizational frameworks. This study explored the utilization of BI methodologies in the identification of potential maternal SSIs after cesarean section procedures, alongside an exploration of the national guidelines governing the management of postcesarean section. The objective of this study is to evaluate the effectiveness of using BI software for monitoring postcesarean infections, with a specific emphasis on antibiotic prescriptions after surgery, and compared the workload between the traditional method and the new approach.

## Methods

### Study setting and design

The diagnostic accuracy study was carried out covering all cesarean section (ICD-9-CM procedures code 74.0, 74.1, 74.2, 74.4, 74.91, and 74.99) performed at Faculty of Medicine Ramathibodi Hospital, Mahidol University, Bangkok, a 1,200-bed tertiary care, from January 1^st^, 2019, through September 30^th^, 2020. Patients who did not present to follow-up within 45 days after the operation were excluded.

### Identification of postcesarean surgical site infections

In this study, the identification of SSIs was carried out by two methods: conventional and postcesarean section infection surveillance systems using BI software.

#### Conventional method

Infection control nurses (ICNs) reviewed all electronic medical records of postcesarean section patients from the time of surgery until 30–45 days after the operation. The diagnosis of SSI was based on the date of event occurring within 30 days after surgery, using the NHSN SSI surveillance definition and used as a gold standard for comparing with another method. The diagnosis of SSI was separately made before obtaining the BI diagnosis.

#### Postcesarean section infection surveillance system using the BI software

A dedicated team of health data analysts employed a BI–based surveillance system to identify SSIs using data captured from routine postoperative follow-up at the outpatient department. Each patient has follow-ups 2 and 4weeks postdischarge. Moreover, some patients who had signs or symptoms of SSIs might visit either the OPD, the emergency department or be admitted to the inpatient ward. These visits and admission information were entered into the hospital’s electronic medical records in a standard fashion. The BI will capture the prespecified criteria among all patients, and patients who have at least one criterion will be considered as potentially having SSI.

These three criteria are as follows:

1. Had one or more of the following ICD-10 codes: *T814*, Complications of procedures not elsewhere classified; *O85*, Puerperal sepsis; *N039,* Other diseases of the urinary system; *N76*, Other inflammation of vagina and vulva; *A049,* Bacterial intestinal infection, unspecified; *N719*, Inflammatory disease of the uterus, unspecified; *N739*, Female pelvic inflammatory disease, unspecified; *N73*, Other female pelvic inflammatory diseases.^5^
2. The use of antibiotics from day 2 to day 30 postsurgery, excluding day 1, which was considered antimicrobial prophylaxis.
3. Microbiology results obtained from day 2 to day 30 postsurgery. Patients with positive culture result on the day of surgery were excluded.

Patients who met at least one of the above three criteria were categorized as potential SSI cases and their medical records were captured by BI. Possible SSI cases were compared with those identified by the conventional method.

### Development of the BI program with a focus on postoperative antibiotic usage (day 8–30 and day 8–45)

Normally, the postoperative infection surveillance protocol spans from day 2 to day 30 after surgery. Alternatively, from our SSI surveillance data, all SSIs cases became apparent after seven days of surgery, therefore, the BI was programmed to detect antibiotics usage from day 8 to day 30 after surgery. Moreover, it was noted that some patients returned after day 30 and reported visiting outside the hospital with clinical features consistent with SSIs. Consequently, we extended our investigation of SSIs by integrating BI to monitor antibiotic utilization from day 8 to day 45 after surgery.

### Sensitivity, specificity, and workload

The conventional method of infection surveillance was used as a reference for calculating sensitivity and specificity in both the initial development and improvement phases of the BI program. The sensitivity and specificity were calculated and compared between the two methods. The sensitivity and specificity were calculated using STATA software (Stata Corp, Collage Station, Tx, USA). The workload was compared between before and after the intervention using the Student’s *t*- test when a normal distribution could be assumed, or the Wilcoxon matched-pairs signed-rank test when a non-normal distribution was present. The normality of continuous variables was assessed using the Shapiro-Wilk test. Nurse workload was quantified in full-time equivalent (FTE) hours by converting the average review time per case into total hours and dividing by the standard 35-hour workweek. FTEs before and after BI implementation were compared to demonstrate the impact on nursing resources. A *P* value of <.05 was considered statistically significant. Statistical analyses were performed using SPSS (IBM Corp., Armonk, NY, USA). This study was approved by the Human Research Ethics Committee of the Faculty of Medicine, Ramathibodi Hospital, Mahidol University (approval number: COA.MURA2022/596).

## Results

### Power BI SSI surveillance system development

In the initial study period from January to September 2019, ICNs monitored 1,501 cesarean section procedures. Within this time frame, potential SSI cases were closely tracked using a BI program, which cross-referenced with our criteria; diagnostic ICD-10 codes, positive microbial cultures, and antibiotic usage. A thorough manual chart review identified 21 cases that met the NHSN criteria for SSI events. In parallel, the BI program identified 405 possible SSIs; 385 patients received antibiotics between day 2 and 30 after surgery, 15 cases were recorded as SSI using the ICD-10 coding system, and 5 patients with positive microbial cultures, respectively. When considered each criterion and compared with gold standard, the antibiotics prescribed in the surveillance period gave the highest sensitivity and specificity as described in Table 1.

**Table 1.** Sensitivity and specificity of SSI cases detection criterion by Power BI program compared to manual chart review (*N* = 1,501)

Variables	No. of Possible SSI Cases by Power BI	No. of Identified SSI Cases by ICN	Senstivity, %	Specificity, %
Microbial cultures positive	**5**	**21**	**24**	**100**
Diagnosed SSI by ICD-10	**15**	**21**	**71**	**100**
Antibiotics prescribed between day 2 and day 30	**385**	**21**	**100**	**75**
Overall criteria	**405**	**21**	**100**	**74**

Note. SSI = Surgical Site Infection; ICN = Infection Control Nurse; Power BI = Business Intelligence software used for surveillance.

### Power BI SSI surveillance system with a focus on postoperative antibiotic usage (Day 8–30 and day 8–45)

One thousand seven hundred forty-two patients were enrolled for the second study period (October 2019 to September 2020). All cases underwent a manual review conducted by ICNs with 39 patients met the SSI criteria. Additionally, the BI program identified 108 potential SSI cases based on antibiotic usage between day 8 and day 30, achieving a sensitivity of 85% and a specificity of 95%. When extending the antibiotic surveillance window to day 8–45, the number of potential SSI cases increased to 145, improving the sensitivity to 97% while maintaining a high specificity of 94% as summarized in Table 2. Furthermore, analysis of the final three months of the study (July–September 2020), which included 452 cases, demonstrated excellent performance of the Power BI surveillance system, with a sensitivity of 100% (95% CI:66.4–100%), a specificity of 93.2% (95% CI:90.5–95.4%), and a ROC area of .966.

**Table 2.** Sensitivity and specificity of SSI cases detection by power BI program focusing on antibiotics usage (*N* = 1,742)

Study period	Day after surgery (ATBs usage)	No. of possible SSI Cases by Power BI	No. of identified SSI Cases by ICN	Sensitivity, % (95% CI)	Specificity, % (95% CI)
Oct 2019- Sep 2020	**Day 8–30**	108	**39**	85 (69.5–94.1)	95 (94.5–96.5)
	**Day 8–45**	145	**39**	97 (89.6–99.6)	94 (92.6–95.2)

Note. ATBs = Antibiotics; SSI = Surgical Site Infection; ICN = Infection Control Nurse; Power BI = Business Intelligence software used for surveillance.

### Workload

In the last three months of the study period (July to September 2020), workload analysis demonstrated that implementing the BI program for postcesarean section infection surveillance led to a significant reduction in cases requiring manual review from 452 to 39 cases (91% reduction).

**Figure 1.**
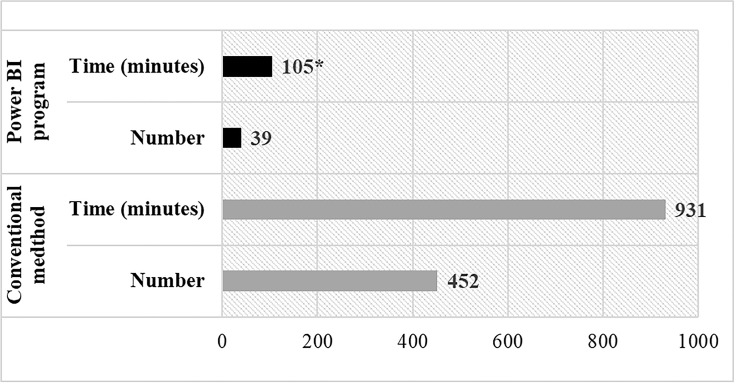
Number of SSI cases and average time-used for monitoring from the conventional method and the power BI program. *P < .001.

Furthermore, the review process took approximately 2 minutes for cases without infections and about 5 minutes for patients with infections. Based on the Wilcoxon signed-rank test, the overall surveillance duration was significantly reduced from 931 minutes to 105 minutes following implementation (*P* < .001), representing an 89% decrease in review time (Figure 1). This reduction saved 826 minutes over three months, decreasing nursing workload from 0.39 FTE/week before implementation to 0.007 FTE/week after, indicating substantial time savings.

## Discussion

Our initial study phase indicated that using only ICD-10 codes or microbial culture results yielded low sensitivity and might not be effective for postcesarean section infection surveillance. This might be attributed to incomplete ICD-10 coding and a small number of postcesarean section patients undergoing microbial culture, which aligned with previous studies on postsurgical gallbladder and colon infections which reported sensitivity of 12.5% and 40.6%, respectively for using the ICD-10 criteria.^6^ On the contrary, a study on postsurgical mastectomy and breast reconstruction, where using infection diagnosis codes gave a sensitivity of 87.5%.^7^ However, when incorporating postsurgical antibiotic usage as a variable in the BI program, we observed an increase in ability to detect possible SSI cases with a sensitivity of 85% (Day 8–30).

Although SSI diagnosis followed the CDC 30-day surveillance criteria, the observation period was extended to better reflect the true incidence. The optimal surveillance window was identified as day 8–45 postsurgery, as most false positives within day 2–7 were due to non-SSI antibiotic use. The BI model within this time frame showed excellent diagnostic performance, with a sensitivity of 100% (and a ROC of .966). Our study is in accordance with a prior study by Bolon MK et al., which employed pharmacy data to enhance postsurgery infection monitoring for total hip arthroplasty (THA) and total knee arthroplasty (TKA). This study found that monitoring antibiotic usage over a period of 7 days or longer after surgery notably enhanced the capacity to identify infections, achieving sensitivity rates of 93% for THA and 86% for TKA respectively.^8^

It was important to highlight that the role of semiautomated SSI surveillance system is increasing.^4^ In our study, we observed this new surveillance method significantly reduced the workload of ICNs by up to 91%, thus allowing for potential expansion to other surgical procedures. The computer-based surveillance system took 9 times less than the manual method. If this task is performed under the conventional method, it would take a large portion of working hours and ICNs would not be able to carry out the comprehensive SSI and other healthcare-associated infection as well as implementation and monitoring of IPC practices within the hospital. This is consistent with the previous study, which indicated that a computer program could effectively reduce the workload in infection surveillance.^4,9^

Nonetheless, there were a few limitations to our study. Firstly, it was conducted in a single tertiary care institution with a well-established follow-up system, which might limit the generalizability of our findings to other settings with less structured postdischarge follow-up. Future studies in multi-center and community hospital settings are warranted to validate the applicability of this model. Secondly, we conducted postsurgical infection surveillance on patients who came for follow-up at the OPD along with their schedules. Patients with SSIs who did not return would be unnoticed. Similar to previous studies, additional tracking methods should be engaged to enhance infection surveillance, such as telephone follow-up and mobile phone reporting.^10,11^ Lastly, the use of BI to capture possible SSIs cannot be applied directly, as all cases flagged by the BI system are subsequently verified by ICNs before reporting to minimize misclassification. However, our study demonstrated the potential benefits of monitoring physician-prescribed antibiotics, helping prevent antibiotic resistance, and improving overall SSI surveillance.

## Conclusions

Surveillance of SSI was essential for maintaining hospital infection prevention standards. Traditional surveillance methods required a significant amount of time and manpower. Using a semi-automated system helped reduce the workload of ICNs, especially for cesarean section. Enhancing surveillance efficiency by focusing on antibiotic usage after surgery during the appropriate time-frame could reduce workload. The new surveillance method should be implemented to the surveillance of SSI in other essential surgeries.
